# The Complete Mitochondrial DNA of *Trypanosoma cruzi*: Maxicircles and Minicircles

**DOI:** 10.3389/fcimb.2021.672448

**Published:** 2021-06-29

**Authors:** Francisco Callejas-Hernández, Alfonso Herreros-Cabello, Javier del Moral-Salmoral, Manuel Fresno, Núria Gironès

**Affiliations:** ^1^ Centro de Biología Molecular Severo Ochoa, Consejo Superior de Investigaciones Científicas, Universidad Autónoma de Madrid, Madrid, Spain; ^2^ Instituto Sanitario de Investigación de la Princesa, Group 12, Madrid, Spain

**Keywords:** kinetoplast DNA, maxicircle, minicircle, strain, *Trypanosoma cruzi*

## Abstract

The mitochondrial DNA of Trypanosomatids, known as the kinetoplast DNA or kDNA or mtDNA, consists of a few maxicircles and thousands of minicircles concatenated together into a huge complex network. These structures present species-specific sizes, from 20 to 40 Kb in maxicircles and from 0.5 to 10 Kb in minicircles. Maxicircles are equivalent to other eukaryotic mitochondrial DNAs, while minicircles contain coding guide RNAs involved in U-insertion/deletion editing processes exclusive of Trypanosomatids that produce the maturation of the maxicircle-encoded transcripts. The knowledge about this mitochondrial genome is especially relevant since the expression of nuclear and mitochondrial genes involved in oxidative phosphorylation must be coordinated. In *Trypanosoma cruzi* (*T. cruzi*), the mtDNA has a dual relevance; the production of energy, and its use as a phylogenetic marker due to its high conservation among strains. Therefore, this study aimed to assemble, annotate, and analyze the complete repertoire of maxicircle and minicircle sequences of different *T. cruzi* strains by using DNA sequencing. We assembled and annotated the complete maxicircle sequence of the Y and Bug2148 strains. For Bug2148, our results confirm that the maxicircle sequence is the longest assembled to date, and is composed of 21 genes, most of them conserved among Trypanosomatid species. In agreement with previous results, *T. cruzi* minicircles show a conserved structure around 1.4 Kb, with four highly conserved regions and other four hypervariable regions interspersed between them. However, our results suggest that the parasite minicircles display several sizes and numbers of conserved and hypervariable regions, contrary to those previous studies. Besides, this heterogeneity is also reflected in the three conserved sequence blocks of the conserved regions that play a key role in the minicircle replication. Our results using sequencing technologies of second and third-generation indicate that the different consensus sequences of the maxicircles and minicircles seem to be more complex than previously described indicating at least four different groups in *T. cruzi* minicircles.

## Introduction

*Trypanosoma cruzi* is a unicellular eukaryotic organism that causes the Chagas disease or American Trypanosomiasis, a chronic endemic illness of Latin America, and a neglected tropical disease (Pérez-Molina and Molina, 2018). Nowadays, it has been estimated that there are around 6–7 million of chronically infected people (World Health Organization). This parasite has a very complex life cycle that includes an invertebrate hematophagous triatomine vector and a broad range of mammalian hosts (Clayton, 2010). Also, *T. cruzi* has different biological stages (de Souza et al., 2010; Rodrigues et al., 2014). The non-infective epimastigotes are present in the midgut of triatomines where they differentiate into infective metacyclic trypomastigotes, that after the infection of host cells are differentiated into the replicative amastigote stages and subsequently into infective trypomastigotes that reach the bloodstream (Echeverria and Morillo, 2019). Chagas disease presents an acute phase with low mortality and symptomatology. Then, the patients can remain in an asymptomatic chronic phase for life or in the 30–40% of them will produce after 10–30 years chronic myocarditis, megavisceras, or both (Rassi et al., 2012).

*T. cruzi* is characterized for showing a great genomic heterogeneity and plasticity across strains (Reis-Cunha et al., 2015; Callejas-Hernández et al., 2018; Herreros-Cabello et al., 2020). This diversity at the genomic level, has promoted the creation of different methods for the classification of hundreds of strains described to date (Zingales et al., 2009; Zingales et al., 2012; Barnabé et al., 2016). These classifications have been established based on some conserved genetic sequences (genomic, mitochondrial and microsatellite DNA). Besides, some researchers have suggested that both the parasite and host genetic variability could be the causes of differential clinical manifestations of Chagas disease (Manoel-Caetano Fda and Silva, 2007). Also, transcriptomic analysis revealed differences between virulent and non-virulent strains (Belew et al., 2017; Oliveira et al., 2020). Indeed, some *T. cruzi* strains highly differ in pathogenicity. Some are acute lethal strains as Y, whereas others, such as VFRA can produce a chronic infection in BALB/c mice (Rodriguez et al., 2014). Furthermore, proteomic analysis, comparing strains with different pathogenicity, indicates that strains inducing chronic infection have enriched antioxidant defenses, while those inducing acute infections produce nucleotides and proteins involved in parasite replication and lethality (Herreros-Cabello et al., 2019).

All the Trypanosomatids have a single large mitochondrion per cell (Maslov et al., 2019). Its mitochondrial DNA is a network of concatenated circular molecules of maxicircles and minicircles that is called the kinetoplast (kDNA). This structure contains dozens of maxicircles (20-40 Kb) and thousands of minicircles (0.5-10 Kb) with varying sizes depending on species (Shapiro, 1993; Lukes et al., 2002; Thomas et al., 2007).

Kinetoplasts may play a role in the pathogenicity of *T. cruzi*, and some researchers suggest the minicircles can integrate into the host genome generating autoimmune events (Simoes-Barbosa et al., 2006; Hecht et al., 2010). Also, maxicircle gene deletions have been associated with asymptomatic patients of Chagas disease (Baptista et al., 2006). Both maxicircles and minicircles have been proposed as targets for molecular detection of *T. cruzi* DNA (Morel et al., 1980; Schijman et al., 2011; Rusman et al., 2019).

Maxicircles contain the characteristic mitochondrial genes of other eukaryotes (Simpson, 1987) and it has been shown that their sequence is characterized by two main regions: the coding region, highly conserved across strains and the divergent/variable region, very difficult to sequence due to its repetitive nature and length variability. Recent findings of maxicircles using third-generation sequencing technologies have revealed that the sequence length may differ across strains, but more importantly, for some strains, its complete sequence is longer than previous estimations (Gerasimov et al., 2020).

Minicircles are exclusive to Trypanosomatids and they are directly involved in U-insertion/deletion editing system as they encode guide RNAs (gRNAs) (Simpson et al., 2003; Aphasizhev and Aphasizheva, 2014; Smith et al., 2020). Moreover, it is suggested that both molecule populations are heterogeneous in the cell, showing strain-specific variations (Westenberger et al., 2006; Messenger et al., 2012).

Several studies have elucidated that the set of minicircles in *T. cruzi* presents a conserved structure among strains of approximately 1.4 Kb, being organized into four highly conserved regions (mHCRs) of 120 bp located 90 degrees apart from each other, and an equal number of hypervariable regions (mHVRs) of 330 bp interspersed between these conserved regions (Leon et al., 1980; Gonzalez, 1986; Macina et al., 1986; Degrave et al., 1988; Avila et al., 1990; Shapiro and Englund, 1995; Guhl et al., 2002; Junqueira et al., 2005). Also, mHVRs diversity have gained attention since they are involved in specific functions that are unique for Trypanosomatids. mHVRs code for gRNAs that direct the edition of several mitochondrial mRNAs converting these primary transcripts into functional messages (Simpson et al., 2003).

The presence of three conserved sequence blocks (CSB) in Trypanosomatids minicircles. CSB-1 (10 bp sequence) and CSB-2 (8 bp sequence) show lower interspecies homology, while CSB-3 (12 bp sequence), known as the Universal Minicircle Sequence (UMS), is conserved within most Trypanosomatids and constitutes the minicircle replication origin (Ray, 1989). Also, the number, size, and location of these CSBs in minicircles seem to differ between species (Ponzi et al., 1984; Sugisaki and Ray, 1987; Degrave et al., 1988; Botero et al., 2018). Concretely, the CSB-3 or UMS is the specific binding site of the UMS binding protein (UMSBP), which has been related with the minicircle replication and kDNA segregation (Milman et al., 2007). UMSBP has been widely studied in *Crithidia fasciculate* (Tzfati et al., 1995; Abu-Elneel et al., 1999; Abu-Elneel et al., 2001; Onn et al., 2006), but its presence has been revealed in other Trypanosomatids such as *T. cruzi* (Milman et al., 2007; Herreros-Cabello et al., 2019), *Leishmania donovani* (Singh et al., 2016) or *T. brucei* (Milman et al., 2007). According to these studies the consensus sequence for each CSB, including *T. cruzi*, would be: CSB-1=AGGGGCGTTC, CSB-2=CCCCGTAC and CSB-3=GGGGTTGGTGTA.

However, a similar analysis is lacking in *T. cruzi*. Therefore, our study aimed to assemble, annotate, and analyze the complete repertoire of maxicircle and minicircle sequences of different *T. cruzi* strains.

## Materials and Methods

### Parasite Cultures and DNA Isolation

Y strain was obtained from Dr. J. David (Harvard Medical School, Boston, Massachusetts, USA), originally isolated back in 1953 (Amato Neto, 2010). Bug2148 strain was obtained from Dr. M. Miles (London School of Hygiene and Tropical Medicine, London, UK) through the European program ChagasEpiNet.

Vero cells were culture with complete Roswell Park Memorial Institute (RPMI, Thermo Fisher Scientific) medium containing 2 mM L-glutamine, 100 UI/ml of antibiotics mixture, 10 μg/m streptomycin and 0.1 mM non-essential amino acids and supplemented with 5% Fetal Bovine Serum (FBS, Gibco Life Technologies, Grand Island, NY) at 37°C in an atmosphere of 5% CO_2_ until the cells reached 80% confluence in biosafety level 3 (BSL3) cell culture laboratories. The cell monolayer was subsequently infected with previously infected Vero cell-derived trypomastigotes (Bug2148 and Y strains). After 4 days, the supernatant medium was collected, dead cells and amastigotes were removed by centrifugation at 1000 g by 5 min and trypomastigotes were collected by centrifugation at 1600 g for 10 min.

DNA from Y strain was isolated using the “High Pure PCR Template Preparation Kit” (Roche). DNA from Bug2148 strain was isolated using the Phenol-Chloroform method to obtain larger fragments for sequencing as needed for PacBio technology. Samples for sequencing were treated with DNAse-free RNAse I (Roche) and quantified by absorbance at 260 nm using the Nanodrop ND-1000 (Thermo Scientific). All samples showed an A260/A280 ratio higher than 2.0 and DNA integrity was assessed by agarose gel electrophoresis.

### Maxicircle and Minicircle Sequencing

DNA from Bug2148 was sequenced using Pacific Biosciences (PacBio) technologies at the Norwegian Sequencing Centre (www.sequencing.uio.no), a national technology platform hosted by the University of Oslo and supported by the “Functional Genomics” and “Infrastructure” programs of the Research Council of Norway and the Southeastern Regional Health Authorities. PacBio library preparation includes a fragment length filtering (>8Kb). DNA from the Y strain was sequenced with Illumina MiSeq series by the Genomics facility at the *Parque Científico de Madrid* (PCM, Madrid, Spain). Both are described in Callejas-Hernández et al. (2018). Integrity from two samples was analyzed in Bioanalyzer (Agilent 2100) to confirm DNA fragmentation level larger than 20 Kb for PacBio and 900 bp for Illumina sequencing. No overlapping Paired-end reads of 2 × 300 format and 8–15 Kb of read length were obtained from Illumina and PacBio, respectively. Raw reads were subject to quality-filtering using standard processes and analyzed using FASTQC tool. Reads with quality lower to 25 (phred score based) and mapped to nuclear genomic DNA references were discarded.

### Assembly and Gene Annotation of *Trypanosoma cruzi* Maxicircle and Minicircle Sequences

Reads from the Y strain were assembled using SPADES (v3.9.0). Bug2148 maxicircle was assembled using HGAP v3 (Pacific Biosciences, SMRT Analysis Software v2.3.0), seed sequence length and minimal coverage values were set to 6 Kb and 15X, respectively. Assembled maxicircles were annotated in a semi-automatic mode. We checked the synteny of maxicircle genes described by Ruvalcaba-Trejo and Sturm (2011) using Blastn searches.

Maxi and minicircle sequences were further tested for circularity, manifested by the presence of directly repeated ends in the assembled molecule. Briefly, contigs were split into two halves, and then Minimus2 [from to Amos (version 3.1.0)] tool was used to identify repeated ends as described by Camacho et al. (2019) and Treangen et al. (2011).

Maxicircle synteny between strains was analyzed using Artemis Comparison Tool (ACT) and R using the genoPlotR package.

### Analysis of Conserved and Variable Motifs in Minicircles

Multiple alignments of the minicircle sequences of Y strain were performed using MUSCLE software (https://www.ebi.ac.uk/Tools/msa/muscle/). The conserved regions of the Y strain minicircles were analyzed using the WebLogo 3 (version 3.7.4) tool (Crooks et al., 2004).

### Validation of Minicircles by PCR Amplification and Sequencing

Experimental validation of some minicircles of Y strain was performed by PCR amplification. Primers were manually designed and then checked *in silico* by Primer Blast, NCBI Blast and TriTrypDB Blast to check the predicted PCR amplicon length and their specificity to *T. cruzi*. Primers for each minicircle are described in **Supplementary Table S1**. The same primers were tested in DNA sample of Bug2148 strain. Amplification was carried out in a 25 µL-final volume using Q5 High-Fidelity DNA Polymerase protocol (New England BioLabs) and 30 ng of DNA. PCR was run on a MyCycler Thermal Cycler (BioRad) using the following profile: initial denaturation at 95°C for 3 min followed by 20 cycles of 30 s at 95°C, 30 s at 58°C and 30 s at 72°C, completed by a final incubation of 5 min at 72°C. Amplification products were analyzed by agarose gel electrophoresis and sequenced using Sanger technology by the *Plataforma de Genómica* of the *Parque Científico de Madrid*.

## Results

### Maxicircles of Y and Bug2148 Strains

In agreement with previous estimations, the total length of the Y strain maxicircle is about 24 Kb. However, in the case of Bug2148 strain, the sequence length is the largest obtained to date with 64.11 Kb, suggesting that an unknown level of complexity may exist for some *T. cruzi* strains. Both maxicircles are available in **Supplementary Data S1, S2**, and accessible in GenBank. However, the complete repertoire of 21 genes listed by Ruvalcaba-Trejo and Sturm (2011) was confirmed in both strains (**Supplementary Table S2**) with minimum sequence length variations (about 15 Kb of the coding sequence in both strains), confirming that the well-documented differences between strains in the maxicircle only depends on the divergent sequence, in agreement to Gerasimov et al. findings (Gerasimov et al., 2020).

**Figure 1:**
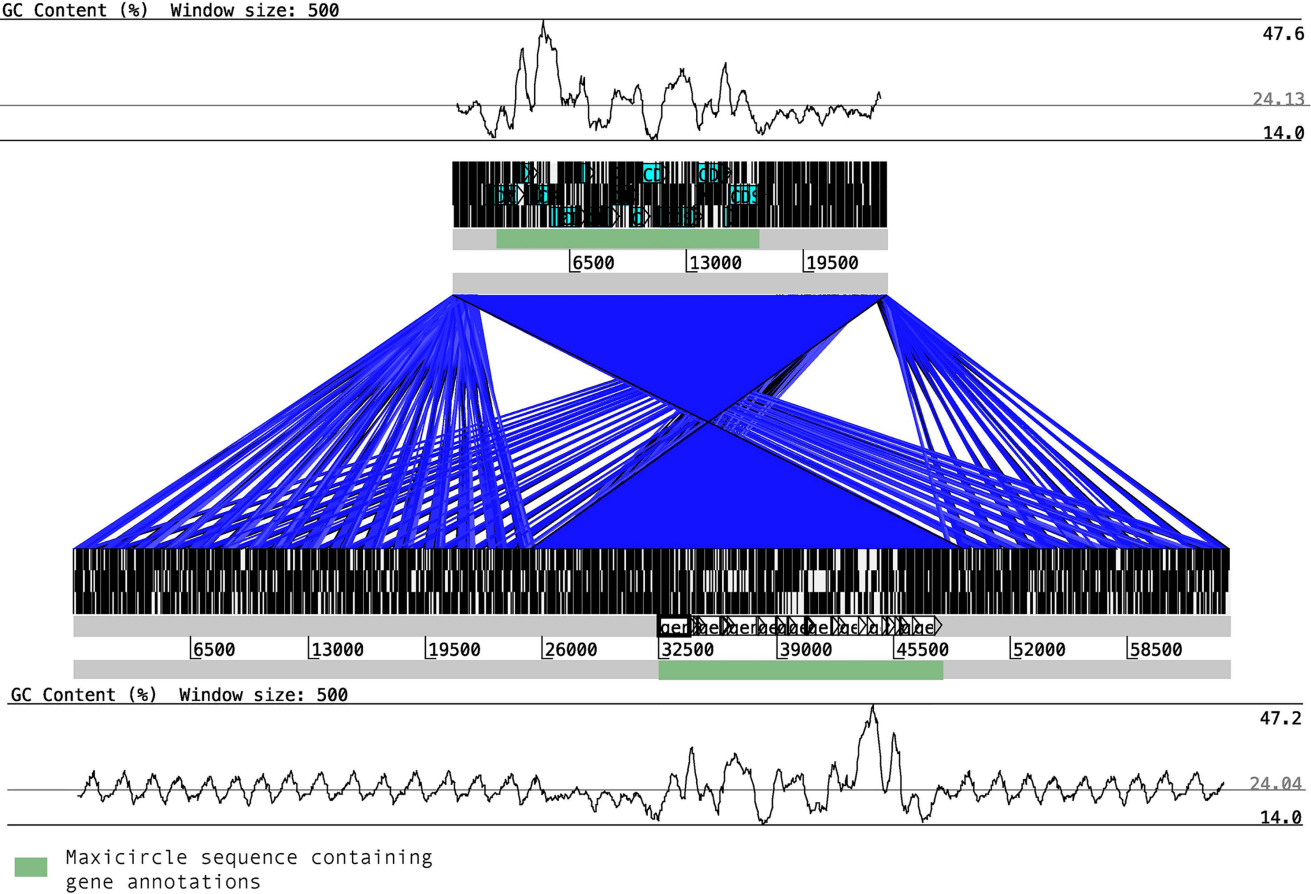
Synteny blocks between *T. cruzi* maxicircles. The maxicircle sequence of Y (upper block) and Bug2148 (low block) are composed of two main regions, the coding region (shown in green boxes) and the variable region, which is mainly composed of short repetitions. The %GC content for each strain shows clear differences between the two main regions.

Synteny between the two maxicircles showed a highly conserved block, corresponding to the coding sequence, perfectly flanked by the variable sequence (**Figure 1**). Besides, for the first time in *T. cruzi*, we found clear differences in %GC content of the two main regions. The variable region is highly enriched with AT (up to 76%) nucleotides while the coding sequences are composed with a more proportional composition of nucleotides (about 47.6% of GC content). Multiple whole maxicircle synteny maps confirm the high conservation of the coding sequence between different *T. cruzi* strains (**Supplementary Figure S1**).

### Validation of *T. cruzi* Minicircles

**Figure 2:**
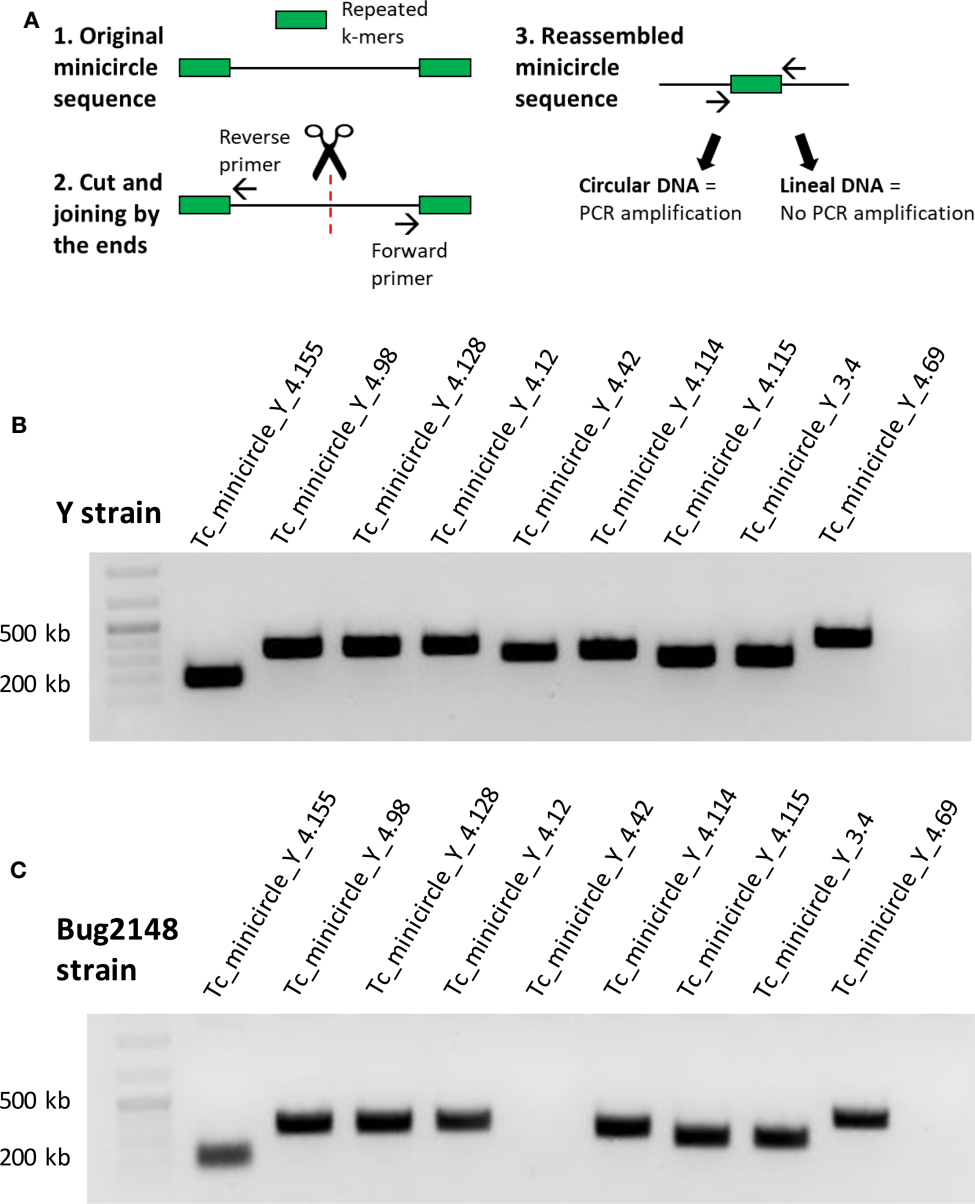
**(A)** Strategy followed to confirm sequence and circularity of the Y strain minicircles. **(B)** PCR products of nine minicircles of the Y strain. **(C)** PCR products with the primers designed against the Y strain, tested in Bug2148 strain.

We were able to assembly minicircle sequences of the Y strain using Illumina (sequences < 1Kb), but it was not possible to assembly Bug2148 strain minicircles using PacBio since library preparation includes a fragment length filtering discarding DNA fragments below 8Kb. We assembled and confirmed the circularity (*in silico*) of 286 different minicircles belonging to the Y strain. Sequences and assembly statistics for all these minicircles are summarized in the **Supplementary Data S3** grouped by size. We designed primers to confirm the sequence and circularity of some of these Y strain minicircles. Primers were designed to amplify a specific band around 200-500 bp (depending on the minicircle total length). The methodology followed to confirm sequence circularity predicted *in silico* is described in **Figure 2A**. We designed primers with opposite directions at 5’ and 3’ ends; in this way, we obtained amplified sequences just for circular and completely assembled minicircles.

We confirmed the sequence and circularity of 9 different minicircles from the Y strain (**Figure 2B**). Sequencing results of the PCR products are shown in **Supplementary Figure S2**. However, we decided to test the same set of primers with the Bug2148 strain (**Figure 2C**). Our results showed one minicircle specific of the Y strain, confirming previous suggestions that the number and sequence of these short molecules may differ between strains.

### Minicircles of Y Strain Display Heterogeneity in Size

Previous studies have proposed that the *T. cruzi* minicircles are highly conserved among strains with a regular size around 1.4 Kb and the same number and length of conserved (mHCR) and variable (mHVR) regions (Degrave et al., 1988; Avila et al., 1990; Guhl et al., 2002; Junqueira et al., 2005).

**Figure 3:**
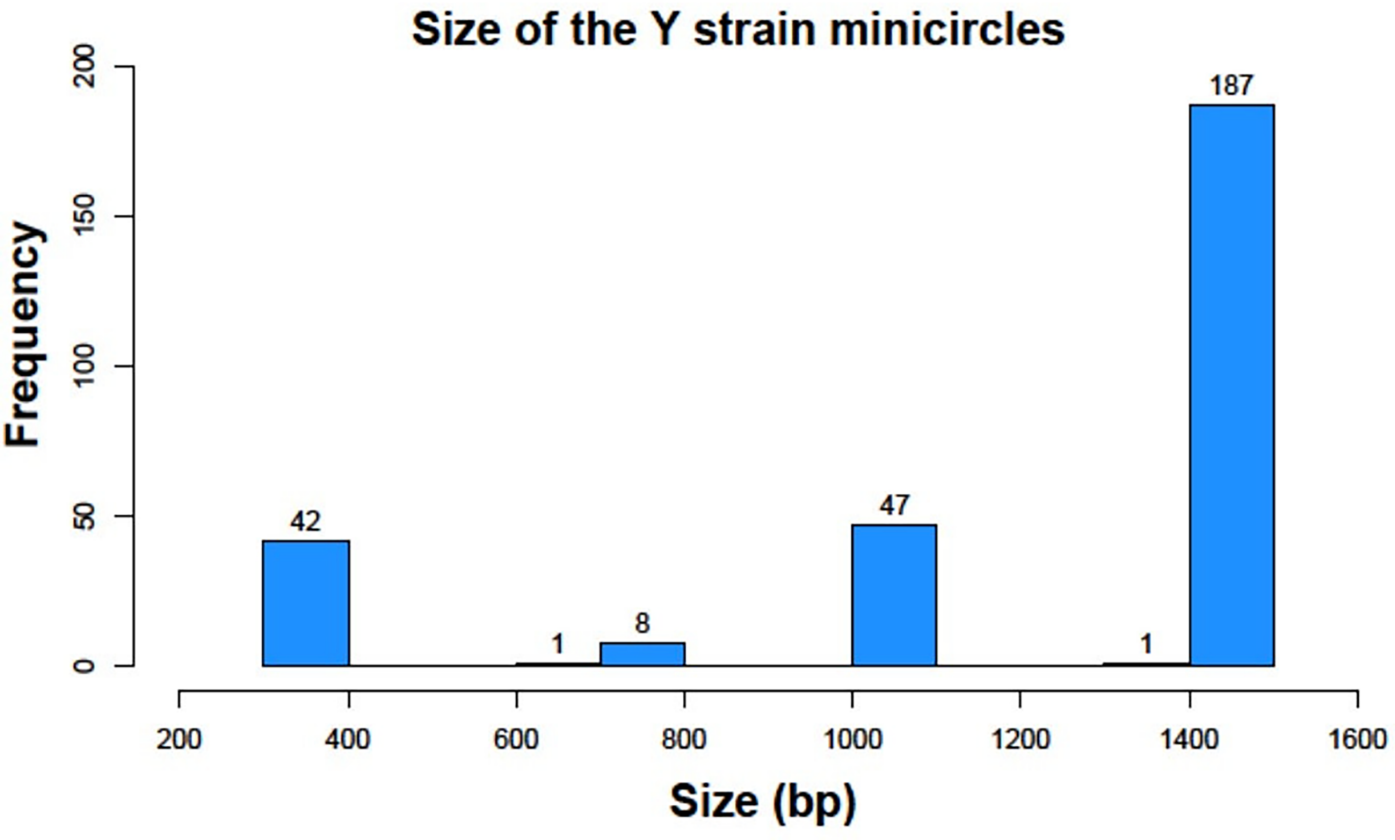
Histogram representation of minicircle sizes of the Y strain in base pairs (bp).

However, despite Y strain was analyzed in these original studies, we have elucidated that different sizes of minicircles exist in this strain with a variable number of mHCRs and mHVRs. Contrary to other Trypanosomatids where the minicircle sequences have a more uniform length, we found that *T. cruzi* minicircles sizes vary from 300 bp to 1400 bp, as we can see in **Figure 3**.

We identified 42 minicircles with sizes between 336-376 bp, the smallest interval. Just 9 minicircles were found between 698-769 bp. 47 had a size between 1051-1095 bp and finally, 188 minicircles belonged to the largest group with 1357-1448 bp This result gives us a clear classification in 4 groups according to the range of sizes, being each group separated from the next one by around 300 bp.

### Analysis of Conserved and Variable Motifs of the Y Strain Minicircle

We analyzed the presence and composition of the mHCRs and mHVRs in the Y strain minicircles. Multiple alignments for each group of size were performed: 336-376 (group 1), 698-769 (group 2), 1051-1095 (group 3) and 1357-1448 bp (group 4).

**Figure 4:**
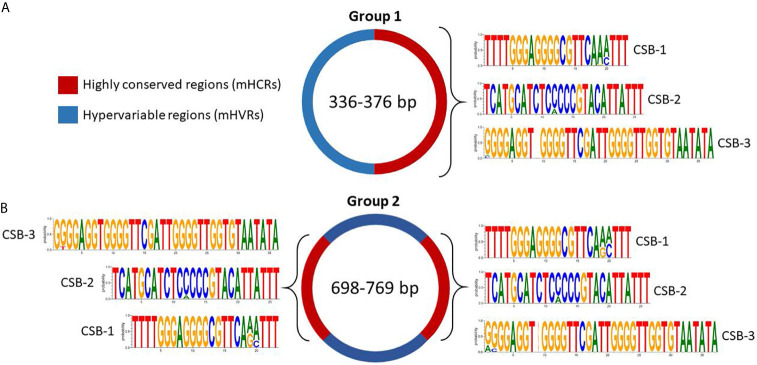
Sequence structure of mHCRs (red) and mHVRs (blue) in group 1 **(A)** and group 2 **(B)** of the Y strain minicircles. Logos of the CSB regions are displayed in each mHCR.

In group 1 all the minicircles present a unique mHCR, and therefore a unique mHVR (**Figure 4A**). CSB-1, CSB-2, and CSB-3 sequences can be seen in the mHCR and the consensus logo of each one is also displayed. Interestingly, in the three CSBs the conserved sequence is longer than the consensus sequence described for Trypanosomatids, suggesting for the first time a larger conserved signature in both ends.

Regarding group 2, the multiple alignment analysis displayed 2 mHCRs and 2 mHVRs (**Figure 4B**). Interestingly, minicircles in group 2 double the size of group 1, and the same occurs with the number of mHCRs and mHVRs. Besides, the logo of each CSB is extremely similar to the respective logo in group 1, showing an increase in the number of conserved nucleotides compared to the theoretical consensus sequence of the *T. cruzi* Y strain CSBs (CSB-1 = AGGGGCGTTC, CSB-2 = CCCCGTAC, CSB-3 = GGGGTTGGTGTA).

**Figure 5:**
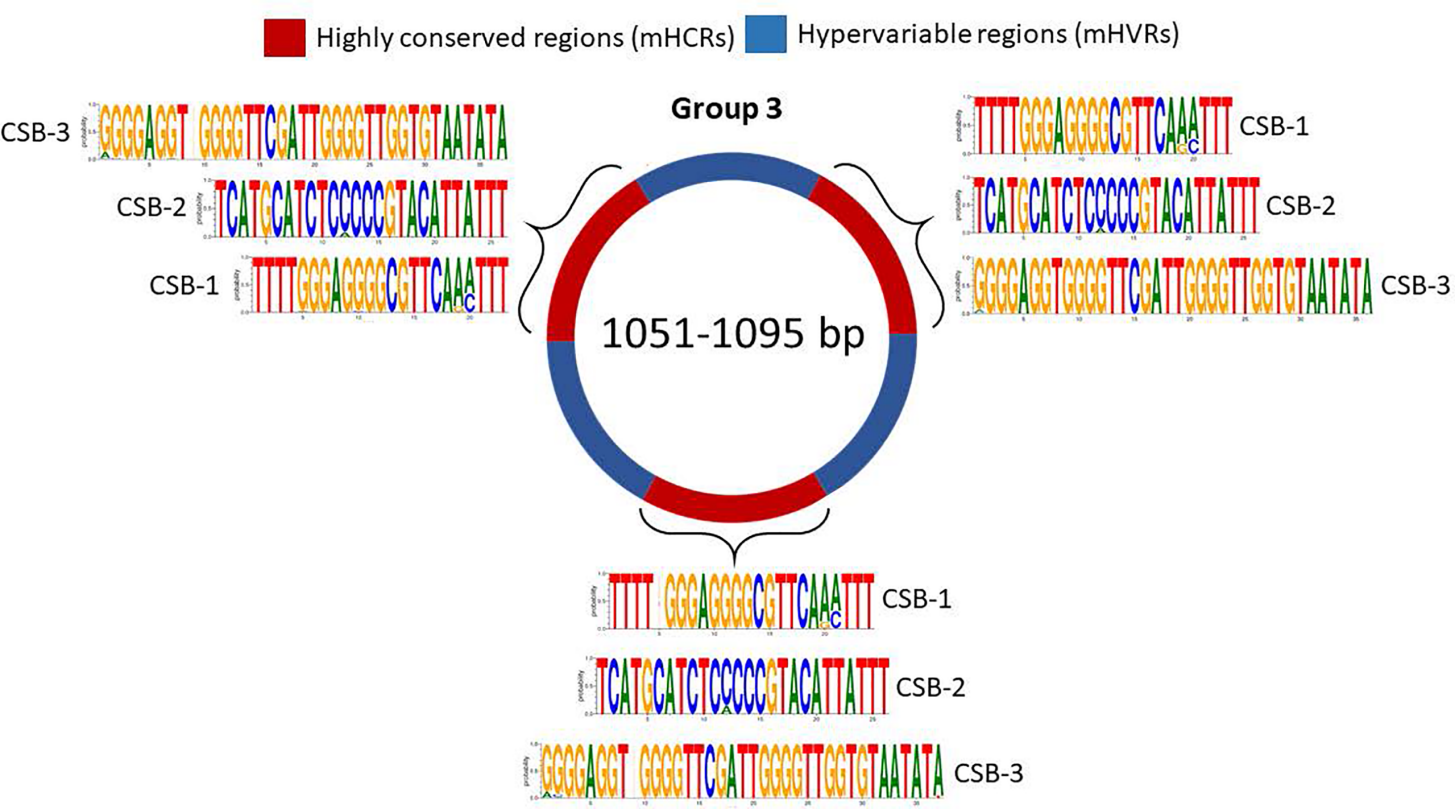
Sequence structure of group 3 minicircles (1051-1095 bp). mHCRs are shown in red and mHVRs in blue. Logos of each CSB region are displayed in each mHCR.

For group 3, we discovered 3 mHCRs and 3 mHVRs in these minicircles (**Figure 5**). Logos of the CSBs showed a similar distribution to those in groups 1 and 2, although logos of the same CSB in each one of these three mHCRs are not exactly the same. There are minimal differences, as the insertion of a nucleotide in a specific minicircle or a variation in the proportion of a nucleotide in a specific position. However, in all the mHCRs the conservation of the principal nucleotide in each position is evident.

**Figure 6:**
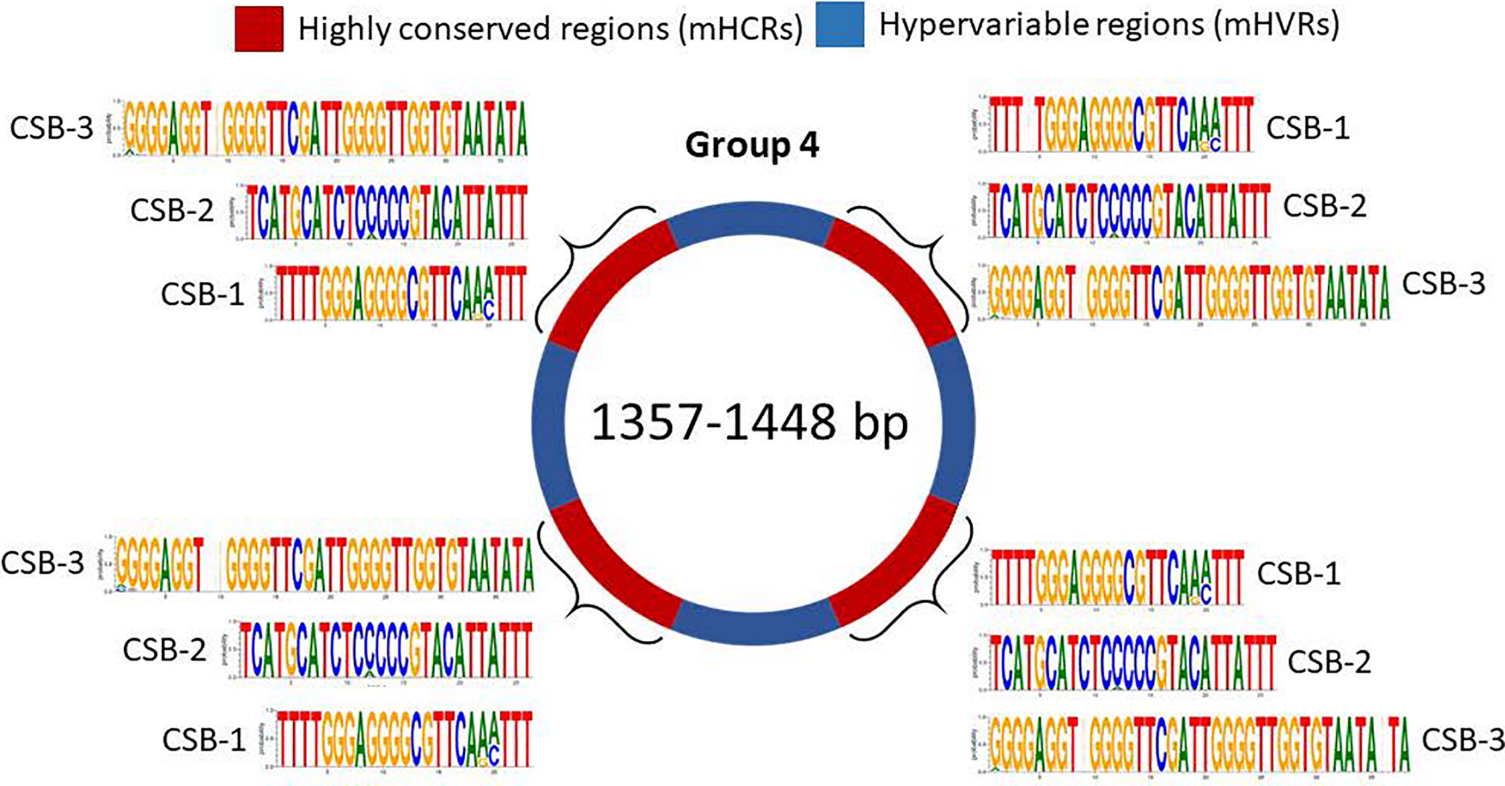
Sequence structure of minicircles from group 4 (1357-1448 bp). mHCRs are shown in red and mHVRs in blue. Logos of each CSB region are displayed in each mHCR.

And finally, minicircles from group 4 contain 4 mHCRs and 4 mHVRs (**Figure 6**). Logos of CSBs were highly similar to the other groups in analysis, although there were specific differences as the insertion of a nucleotide or changes in the nucleotide proportion in some positions.

Interestingly, each group in the analysis had an increment of around 300 bp in size with respect to the immediately shorter. According to our results, it seems like the minicircle needs a new mHCR each 300 bp. Minicircles in group 1 have only a mHCR, while minicircles in group 4 have four mHCRs, one every 90 degrees in the circular structure as it has been well documented (Degrave et al., 1988; Junqueira et al., 2005). Also, in all the minicircle groups the consensus sequence of each CSB seems to be longer than those previously described (Botero et al., 2018).

## Discussion

We report relevant information regarding maxicircles and minicircles kinetoplast DNA isolated from cell-derived parasites. Using second and third generation sequencing techniques we unraveled more complexity than previously reported. Being aware of the limitations of dealing with this complex parasite we were able to obtain valuable results by comparing data from different technologies.

Contrary to the first maxicircles assembled and described in *T. cruzi* (Westenberger et al., 2006), our results suggest that their total length may be larger than previous suggestions, at least for some strains as one analyzed here. Interestingly, this work indicated that the mitochondrial genes are highly conserved between strains and the coding region is about 16 Kb long, independently of the maxicircle length. The main difference between strains (at maxicircle level) corresponds to the variable/divergent sequence length, which also does not have conserved motifs or repetitive patterns. Besides, clear differences in %GC content between the coding and variable regions were found, suggesting, on one hand, the importance of the maxicircle stability and structure and on the other hand, that the transcription efficiency in *T. cruzi* is highly correlated with a proportional sequence composition.

We detected a total of 286 different minicircles. This is the first time that the complete repertoire of different minicircles of a particular *T. cruzi* strain is described. The general structural organization of some *T. cruzi* minicircles was described (Degrave et al., 1988; Avila et al., 1990; Guhl et al., 2002; Junqueira et al., 2005). Their common scheme would be a minicircle with a size around 1.4 Kb, in which four mHCRs of 120 bp are interspersed with four 330 bp regions that would correspond to the mHVRs. However, our results suggest that this organization, at least in some strains, is more complex, suggesting a new potential aggrupation in at least four main groups of minicircles according to their size and the number of mHCRs and mHVRs: 336-376 bp (group 1), 698-769 bp (group 2), 1051-1095 bp (group 3) and 1357-1448 bp (group 4). Minicircles of group 1 have only one mHCR and mHVR. Group 2 minicircles have two mHCRs and mHVRs. Those belonging to group 3 present three mHCRs and mHVRs. And finally, minicircles of group 4 have four mHCRs and mHVRs. The most abundant was group 4, with 188 members, and the less abundant the group 2 with just 9.

The circularity of minicircles was validated by PCR amplification using specific primers of different groups that amplified the common regions detected at both 3’ and 5’ ends of the reads. Primers were designed for the Y strain, but were also amplified in the Bug2148 strain, except for one set of primers, suggesting that these minicircles may be also present in this strain.

Interestingly, the four groups we have described here in Y strain differ in size by about 300 bp, and this distance is always conserved between the mHCRs in all the groups. Also, the size of the mHCRs is around 120 bp in all of the groups. Therefore, the common patterns of distance are the same as those previously described, which may explain the number of mHCRs depending on the size of the minicircle. Previous analysis detected minicircles around 1.4 Kb (Degrave et al., 1988; Avila et al., 1990; Guhl et al., 2002; Junqueira et al., 2005), as those ascribed to our group 4 (the group with more members). Thus, the new techniques of Next Generation Sequencing used here have likely improved the detection of all minicircles regardless of their abundance.

Also, we analyzed the presence and number of the CSB-1, CSB-2, and CSB-3 in the mHCRs of the Y strain minicircles. Previous studies had determined a consensus sequence for each CSB in other Trypanosomatids, although little differences in length and nucleotide composition have been described between species for the CSB-1 and CSB-2 (Barrois et al., 1981; Jasmer and Stuart, 1986; Nasir et al., 1987; Botero et al., 2018). However, in our study of the Y strain of *T. cruzi* we have highlighted that this theoretical consensus sequence for *T. cruzi* is larger than the previous one, with more conserved nucleotides in both ends in all the CSBs of the Y strain minicircles. Interestingly, there are even small differences as changes in the nucleotide proportion in a position or nucleotide insertions between mHCRs of distinct minicircle groups and between the mHCRs of the same minicircle group.

For decades, many researchers have used for their experiments this standard organization of 1.4 Kb and 4 mHCRs and 4 mHVRs of the *T. cruzi* minicircles, although sequences in the NCBI databases of some minicircles suggest a variety of sizes between less than 100 bp and more than 1000 bp. However, the vast majority of submitted sequences correspond to unpublished data, partial or only the hypervariable regions of specific minicircles sequences (Telleria et al., 2006; Velazquez et al., 2008). Therefore, our contribution with the new full complement of different 286 minicircles of the Y strain supposes a great advance in the knowledge and current data that we have of this type of circular structures.

While *T. cruzi* has four groups of minicircles, other species as *Trypanosoma rangeli* (*T. rangeli*) display three different classes of minicircle sequences that are known as KP1, KP2, and KP3 according to the number of conserved regions. They may present one conserved region (KP1), two conserved regions located at 180 degrees (KP2), or four conserved regions, located at 90 degrees (KP3) ( (Recinos et al., 1994; Vallejo et al., 2002). However, while in *T. cruzi* the size of the minicircles varies between 336 and 1448 bp, *T. rangeli* displays a less size variation in minicircles with a range of 1.6-1.8 Kb (Vallejo et al., 1994). In *Trypanosoma copemani*, minicircles have 2048 bp of size and two classes were suggested considering the number of conserved and variable regions: the G1M1 minicircles with two conserved and two variable regions, and the G1M2 minicircles with four conserved and four variable regions (Botero et al., 2018).

In *Leishmania major* 97 different minicircles were detected, and 49 in *Leishmania infantum*, although in these species the size of their minicircles is very uniform, between 660-876 bp and 775-832 bp respectively (Camacho et al., 2019), while *T. cruzi* presents a bigger size minicircle heterogeneity. Moreover, the researchers described the minicircles of *Leishmania* with only one conserved region, while for the same minicircle size *T. cruzi* displays two, and the CSBs present differences in sequence length and nucleotide composition between both species. Taking into account that these conserved regions contain the replication origin, it seems that *Leishmania* only needs one to perform the completed minicircle replication, while *T. cruzi* needs one each 300 bp, probably due to the different efficiency in this biological process between both Trypanosomatids.

Mitochondrial metabolism and gene expression are highly regulated to deal with all those complicated environmental changes across the complete life cycle alternating between the mammalian host and insect vector, including regulation of mRNAs that require extensive uridine insertion/deletion (U-indel) editing for their maturation, as has been described in other closely related Trypanosomatids (Smith et al., 2020). To our knowledge, it is the first time describing a large repertoire of complete *T. cruzi* minicircle sequences.

We sequenced DNA from cell-derived trypomastigotes while in previous reports used epimastigotes. It could be argued that kinetoplast DNA may change from one stage to another. According to Riou and Gutteridge (1978), there should not be differences in DNA content between different stages of *T. cruzi*. On the other hand, Schwabl et al. (2019) found genomic changes over the same stage just by culture passage. Thus, it would be interesting to further investigate whether the increase in complexity could be due to changes in DNA content between epimastigotes and trypomastigotes.

Finally, considering that there are minicircles conserved among strains and their relevant role for the maturation of the maxicircle-encoded transcripts, critical for the replication and survival of the parasite, their analysis may contribute to the understanding of the mitochondrial transcription and translation processes potentially related to the Chagas disease.

## Data Availability Statement

The complete maxicircle sequences for Y and Bug2148 strains are available from the Genbank database accession numbers MW732647, and MW732648, respectively.

## Author Contributions

FC-H, MF, and NG conceived the idea, MF and NG acquired funding. FC-H, AH-C, and JM-S performed the experiments. FC-H and AH-C wrote the first version of the manuscript. All authors reviewed and edited the manuscript. All authors contributed to the article and approved the submitted version.

## Funding

This work was supported by: “Ministerio de Economía y competitividad” and “Fondo Europeo de Desarrollo Regional” (SAF2015-63868-R (MINECO/FEDER) to NG, and SAF2016-75988-R (MINECO/FEDER) to MF); “Ministerio de Ciencia, Innovación y Universidades-Agencia Estatal de Investigación” and “Fondo Europeo de Desarrollo Regional” (PGC2018-096132-B-I00 (MICINN/FEDER) to NG); “Red de Investigación de Centros de Enfermedades Tropicales” (RICET RD12/0018/0004 to MF); Comunidad de Madrid (S-2010/BMD-2332 to MF); FC-H was recipient of a Ph.D. studentship number 411595 (“Consejo Nacional de Ciencia y Tecnología” (CONACYT, Mexico) and the “Consejo de Ciencia, Tecnología e Innovación de Hidalgo” (CITNOVA, Mexico)); AH-C was recipient of a FPU contract of the “Ministerio de Ciencia, Innovación y Universidades”; JM-S was recipient of a “Garantía Juvenil” predoctoral fellowship (Comunidad de Madrid); and Institutional grants from “Fundación Ramón Areces” and “Banco de Santander”.

## Conflict of Interest

The authors declare that the research was conducted in the absence of any commercial or financial relationships that could be construed as a potential conflict of interest.
