# Supplementary material for: The Complete Mitochondrial DNA of Trypanosoma cruzi: Maxicircles and Minicircles
Source: Front Cell Infect Microbiol. 2021 Jun 29;11:672448. doi: 10.3389/fcimb.2021.672448 (PMC8277381; doi:10.3389/fcimb.2021.672448)

Supplementary Figure S1. Multiple whole maxicircle synteny maps confirming the high conservation of the coding sequence between different *T. cruzi* strains: Dm28c, TCC (Gerasimov et al., 2020), Y and Bug2148.

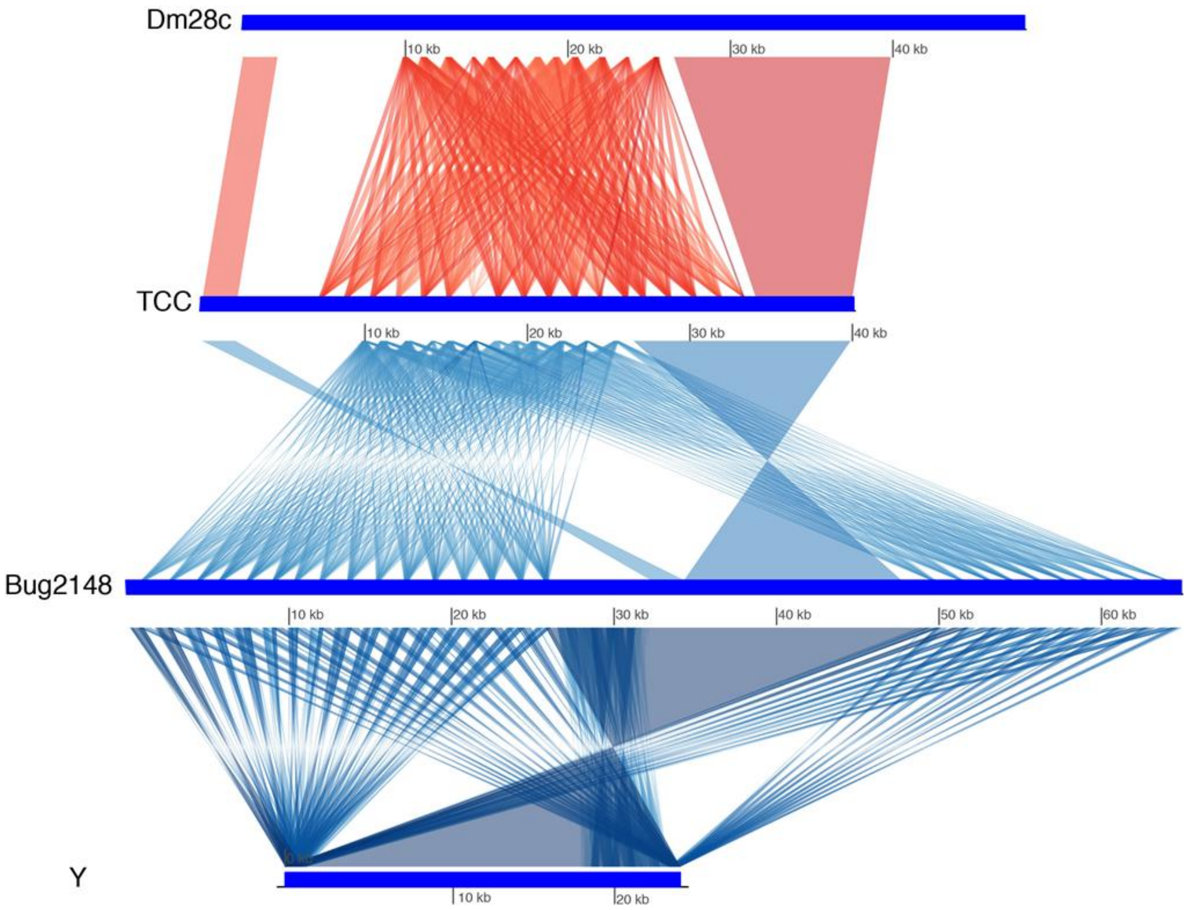

Supplement: Supplementary file 4 [file Presentation_1.pdf]
