# Supplementary material for: The Complete Mitochondrial DNA of Trypanosoma cruzi: Maxicircles and Minicircles
Source: Front Cell Infect Microbiol. 2021 Jun 29;11:672448. doi: 10.3389/fcimb.2021.672448 (PMC8277381; doi:10.3389/fcimb.2021.672448)
Supplement: Supplementary file 5 [file Presentation_2.pdf]

Supplementary Figure S2. Alignment of the sequence of each one of the nine Y strain minicircles tested by PCR with the sequencing result in red. In all the cases the central zone was amplified confirming the circular structure of these minicircles.

Tc_minicircle_Y_4.155

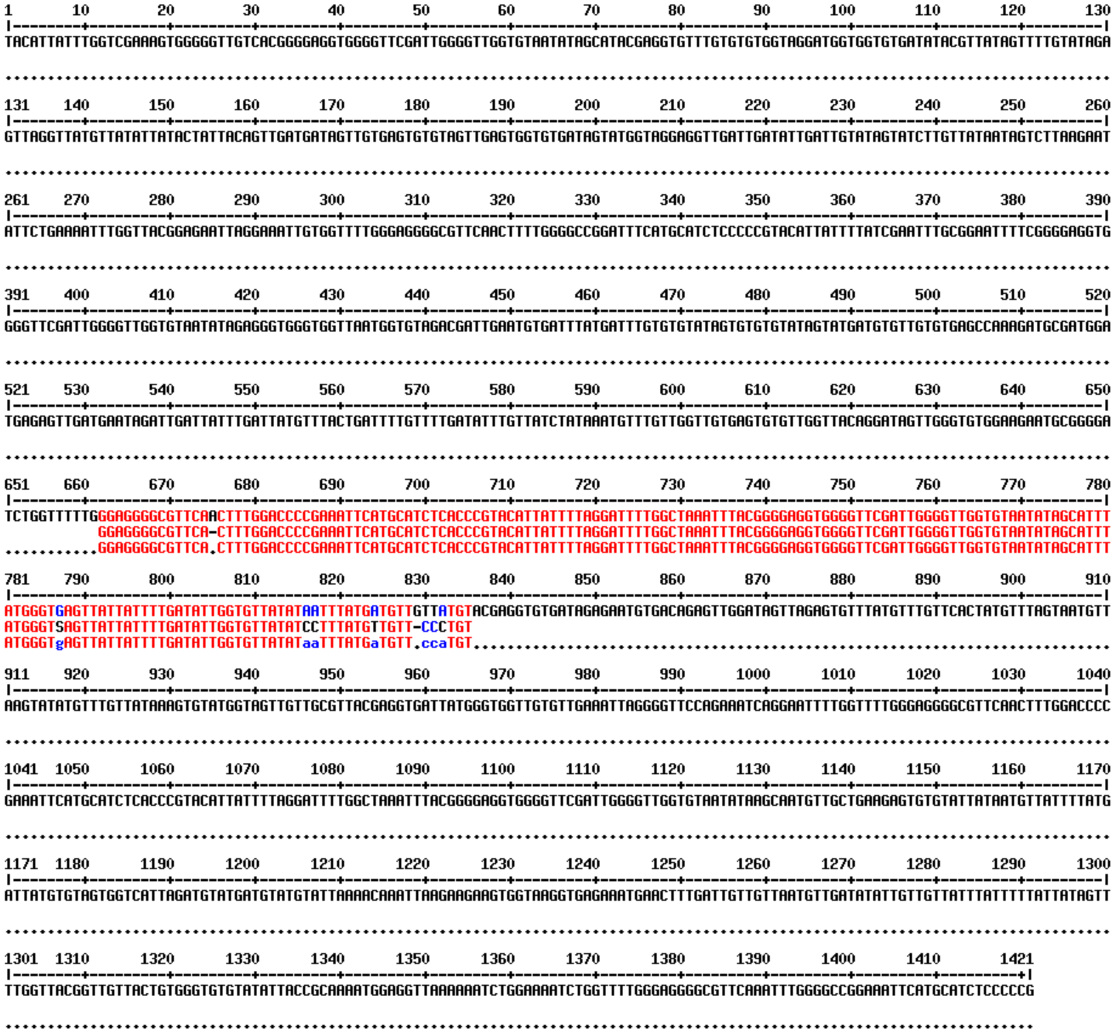

Tc_minicircle_Y_4.98

```
1       10        20        30        40        50        60        70        80        90        100       110       120       130
|--------+---------+---------+---------+---------+---------+---------+---------+---------+---------+---------+---------+---------|
ACATTATTTGGCTGAAAATGGGGGTTGTCACGGGGAGGTGGGGTTCGATTGGGGTTGGTGTAATATAGAATGTGTGTAGGTATGGGTGTGGTCTATGATTTGTATTTTGTGTGTTGTGTATTAGTGATTA

..................................................................................................................................
131     140       150       160       170       180       190       200       210       220       230       240       250       260
|--------+---------+---------+---------+---------+---------+---------+---------+---------+---------+---------+---------+---------|
TGGTAGATTATGCAGTCAAGATATCGATGAAGAGTTATAGTTGATAATTGGTATAGAATGTTTGTGAATGTTTTATTGTTATTAATCTTATACTGTATGAGGGGTTATTGTTGGTGAGGTGGGTGTGTGG

..................................................................................................................................
261     270       280       290       300       310       320       330       340       350       360       370       380       390
|--------+---------+---------+---------+---------+---------+---------+---------+---------+---------+---------+---------+---------|
TTTATTAAGGGTTAAATCGGGGTATAAAATTCGTGGGACTTTGGTTTTGGGAGGGGCGTTCAACTTTTGGGCCGCGATTTCATGCATCTCCCCCGTACATTATTTTGGTCATTTTGTGATTTTGGCGGGG

..................................................................................................................................
391     400       410       420       430       440       450       460       470       480       490       500       510       520
|--------+---------+---------+---------+---------+---------+---------+---------+---------+---------+---------+---------+---------|
AGGTGGGGTTCGATTGGGGTTGGTGTAATATAGGCTTAGGTGGAGGAGGGGTTGATTATGTTATTATTGTTTTATAGTAATGGATCTTGGATGCGATAGTTATGACGATAGATAGAAACAAGATGTGCGT

..................................................................................................................................
521     530       540       550       560       570       580       590       600       610       620       630       640       650
|--------+---------+---------+---------+---------+---------+---------+---------+---------+---------+---------+---------+---------|
GGATAAGAGATAGAGATGGTGCTCTTTGATTGTTTAGGATAGATTTGTTATAACCTCTATTGTATGATGATTGTAATATGGTTACGGTGTTGGTGTATGCTGTGGTAAATTTGGGTTCTGAAAATTCGGG

..................................................................................................................................
651     660       670       680       690       700       710       720       730       740       750       760       770       780
|--------+---------+---------+---------+---------+---------+---------+---------+---------+---------+---------+---------+---------|
AATCTTGGTTTTGGGAGGGGCGTTCAAATTTGGGGCCGGAAATTCATGCATCTCCCCCGTACATTATTTTGTCAAAATTGGGGGTTGTCACGGGGAGGTGGGGTTCGATTGGGGTTGGTGTAATATAGGC
                  GGCGTTCAAATTTGGGGCCGGAAATTCATGCATCTCCCCCGTACATTATTTTGTCAAAATTGGGGGTTGTCACGGGGAGGTGGGGTTCGATTGGGGTTGGTGTAATATAGGC
..................GGCGTTCAAATTTGGGGCCGGAAATTCATGCATCTCCCCCGTACATTATTTTGTCAAAATTGGGGGTTGTCACGGGGAGGTGGGGTTCGATTGGGGTTGGTGTAATATAGGC
781     790       800       810       820       830       840       850       860       870       880       890       900       910
|--------+---------+---------+---------+---------+---------+---------+---------+---------+---------+---------+---------+---------|
AGAAGGTGGTTTAGAAATTTTGTTTATTGTATTGGTAAGAAATGATTTATATGTTCAGTTGACTAGAGGATAATTTTGTTTTGTGAGATGTTAGTTAGTTAGTTAGTTAATCAGTTAAAGTTGTAGAATA
AGAAGGTGGTTTAGAAATTTTGTTTATTGTATTGGTAAGAAATGATTTATATGTTCAGTTGACTAGAGGATAATTTTGTTTTGTGAGATGTTAGTTAGTTAGTTAGTTAATCAGTTAAAGTTGTAGAATA
AGAAGGTGGTTTAGAAATTTTGTTTATTGTATTGGTAAGAAATGATTTATATGTTCAGTTGACTAGAGGATAATTTTGTTTTGTGAGATGTTAGTTAGTTAGTTAGTTAATCAGTTAAAGTTGTAGAATA
911     920       930       940       950       960       970       980       990       1000      1010      1020      1030      1040
|--------+---------+---------+---------+---------+---------+---------+---------+---------+---------+---------+---------+---------|
GATATTAATAATTTGATGTGGTGGTTATGGTTACTGTTTGTTGTTTGTGTATGTGATAATAGCTGGGTAACGGTAAATTTATGGTTTTAAAATTCGGGAAAGTTGTGTTTTGGGAGGGGCGTTCAACTTT
GATATTAATAATTTG
GATATTAATAATTTG...................................................................................................................
1041  1050      1060      1070      1080      1090      1100      1110      1120      1130      1140      1150      1160      1170
|--------+---------+---------+---------+---------+---------+---------+---------+---------+---------+---------+---------+---------|
TGGGCCGCGATTTCATGCATCTCACCCGTACATTATTTTGCCGATTTTGCTGGATTTTCGGGGAGGTGGGGTTCGATTGGGGTTGGTGTAATATAGAGTCGAGGTGGTGTATTGGGTAATGGAGTTTTAT

..................................................................................................................................
1171  1180      1190      1200      1210      1220      1230      1240      1250      1260      1270      1280      1290      1300
|--------+---------+---------+---------+---------+---------+---------+---------+---------+---------+---------+---------+---------|
TGTAGTTGATTATGTTGTAGATGTATGTGATTGATGTTGTTATACATGTGAGTATATGATGTGTAGAACGTTGAGATAGTTTAAGTAGTGTATTGTTTATAGTGAAGTATATTGTACTGTATAATTAAGA

..................................................................................................................................
1301  1310      1320      1330      1340      1350      1360      1370      1380      1390      1400      1410      1420    1428
|--------+---------+---------+---------+---------+---------+---------+---------+---------+---------+---------+---------+-------|
TGGTTTAGGTATGGTTGGAGTGTGAATGGTTACGGGTATAGTTAAAATATGGTTTCGGAAAATCGTGAAAATTCGATTTTGGGAGGGGCGTTCAAATTTGGGGCCGCAATTTCATGCATCTCCCCCGT

................................................................................................................................
```

Tc_minicircle_Y_4.128

```
1        10        20        30        40        50        60        70        80        90        100       110       120       130
AAAATAATGTACGGGGGAGATGCATGAATTTCCGGCCCAAAATCTGAACGCCCCTCCCAAAACCAACTTTCCACGAATTTCCAAACCCAATTTTTATCACAAAGCATGCACTACCATATCACTAACCCGT

131      140       150       160       170       180       190       200       210       220       230       240       250       260
TAAACATTATTTCGAAACATCATCATAGAATACATAACAAACTACACCAAACATATGCTCAACTATCTCTCACCACCACAACACACAATAACTATCACAACAAACATAACAAACATAACATAACATACTA

261      270       280       290       300       310       320       330       340       350       360       370       380       390
TACACCAATAAATCAAGTATACCACTACCTACCGCCCATCCCTATATTACACCAACCCCAATCGAACCCCACCTCCCCGTGACAACCCCCAAAAATCCCAAAATAATGTACGGGGGAGATGCATGAATTT

391      400       410       420       430       440       450       460       470       480       490       500       510       520
CCGGCCCAAAATCTGAACGCCCCTCCCAAAATCAAATTTTCCGCTAATTTTTATACCTCCATATCAACCACAGTAACAGTATACCACCTACACCAATAACCTAAACCAAACTCTAAAACAAAATATAACA

521      530       540       550       560       570       580       590       600       610       620       630       640       650
ATAATAAACAAGTATACATCTACTATACAAAACAATCAAACATTACTCACGCACAAATCTATTCATACACGTTCTCTACCACATACTAAACAAATCTAAACAAATCTATACAAACAAGAAACCATATTCT
                                                                                                                        AAACCATATTCT
                                                                                                                        AAACCATATTCT

651      660       670       680       690       700       710       720       730       740       750       760       770       780
GTCTATATTACACCAACCCCAATCGAACCCCACCTCCCCGTTAAAATCCACAAAATCACGGAAATAATGTACGGGTGAGATGCATGAATTTCCGGCCCAAAATCTGAACGCCCCTCCCAAAACCGAATTT
GTCTATATTACACCAACCCCAATCGAACCCCACCTCCCCGTTAAAATCCACAAAATCACGGAAATAATGTACGGGTGAGATGCATGAATTTCCGGCCCAAAATCTGAACGCCCCTCCCAAAACCGAATTT
GTCTATATTACACCAACCCCAATCGAACCCCACCTCCCCGTTAAAATCCACAAAATCACGGAAATAATGTACGGGTGAGATGCATGAATTTCCGGCCCAAAATCTGAACGCCCCTCCCAAAACCGAATTT

781      790       800       810       820       830       840       850       860       870       880       890       900       910
TCCGAAATTTTTAACCCCATTTTCTTATGGAACCACAACACAAGCACACCCTACCCAACCATTAAATATTCATACAATACATAACTAAGTAACATAGTAACATTAAACTTATTAACTTGACCACTACAAC
TCCGAAATTTTTAACCCCATTTTCTTATGGAACCACAACACAAGCACACCC
TCCGAAATTTTTAACCCCATTTTCTTATGGAACCACAACACAAGCACACCC

911      920       930       940       950       960       970       980       990       1000      1010      1020      1030      1040
TTTACACCATCTCAATCTCTACGACTACACACATCAATACTTAATCTCAGACAAACAAATACTATTCAACAAATAACAAATCTAAACCATTATCCACACCTATGTATATATTACACCAACCCCAATCGAA

1041  1050       1060      1070      1080      1090      1100      1110      1120      1130      1140      1150      1160      1170
CCCCACCTCCCCGTGACAACCCCCTAATTTCGCAAAATAATGTACGGGGGAGATGCATGAATTTCCGGCCCAAAATCTGAACGCCCCTCCCAAAACCAAACTTCCACCAATCTTCCGTAGCCTATCTTAT

1171  1180       1190      1200      1210      1220      1230      1240      1250      1260      1270      1280      1290      1300
CAAGTTCAAACCACTCCAAACATAACAATATAATATAATCAAATAAATACACCTACATAATTTCGATAACAAACTCTTACAACTACAATCTCAAAACACTAGTTCTCCGTCACTAACATATCACAACTAT

1301  1310       1320      1330      1340      1350      1360      1370      1380      1390      1400      1410      1420
ACATCTTAAACTAACCAATACAAAATCTACTTTCAAATTCATAAACCATTACCTCACGCACACTATATTACACCAACCCCAATCGAACCCCACCTCCACGTGACAACCCCCTAAAATCAC
```

Tc_minicircle_Y_4.12

```
1        10        20        30        40        50        60        70        80        90        100       110       120       130
AAATAATGTACGGGGGAGATGCATGAAAATCCGACCCAAAAGTTGAACGCCCCTCCCAAAACCAGAATTTCCGATTTACCGCAACCACATTTTCAGGATATTTTAAGAAACAAAGAATACTATGCTACAA

131      140       150       160       170       180       190       200       210       220       230       240       250       260
AATCATAATCAAATTCATAATCTATTACACACCCTTATCGCTTAACCATGAATCTAAACATCAAATAACATAAATAACAAAATCTATTCAACCTAAACTCACTCCATCTGTAACCATACACAAACTATCA

261      270       280       290       300       310       320       330       340       350       360       370       380       390
CATAACTAAATCACAACTGTGCTACCATAATCCACACCATCAATCAGATGCTATATTACACCAACCCCAATCGAACCCCCACCTCCCCGAAAATTCAGCAAATTCCGCAAAATAATGTACGGGGGAGATG

391      400       410       420       430       440       450       460       470       480       490       500       510       520
CATGAATTTCCGGTCCCAAATTTGAACGCCCCTCCCAAAATCAAGTTTCTCAGTTTTCAGAACCCACAAATTTACTGCAACCCACTAACAAACAACCCATTAACCTAACACACATAAATAACAATAACAT

521      530       540       550       560       570       580       590       600       610       620       630       640       650
AATAGACTACCATTATCACATTTAAACAACACAAACATCTCAACCTATCCAACTTTATATTCTGTGGTAAACATATATCACACTATAACATAAGTCAACCAAATAAAACATAACCGATTAAATATTACTA
                                                                                                     AATAAAACATAACCGATTAAATATTACTA
                                                                                                     AATAAAACATAACCGATTAAATATTACTA

651      660       670       680       690       700       710       720       730       740       750       760       770       780
CCTCAACCCCGCCTATATTACACCAACCCCAATCGAACCCCACCTCCCCGCAAAAATCCACAAATTGACCGAAATAATGTACGGGGGAGATGCATGAAATCTGGCCCCCAAATTTGAACGCCCCTCCCAA
CCTCAACCCCGCCTATATTACACCAACCCCAATCGAACCCCACCTCCCCGCAAAAATCCACAAATTGACCGAAATAATGTACGGGGGAGATGCATGAAATCTGGCCCCCAAATTTGAACGCCCCTCCCAA
CCTCAACCCCGCCTATATTACACCAACCCCAATCGAACCCCACCTCCCCGCAAAAATCCACAAATTGACCGAAATAATGTACGGGGGAGATGCATGAAATCTGGCCCCCAAATTTGAACGCCCCTCCCAA

781      790       800       810       820       830       840       850       860       870       880       890       900       910
AACCAAACTTCCACGATTTTCCGTAACCATAAATTGATGAATAATGTTCTAATACACAAAACAGAATAAACTGATAACAACAAATCTATCACATAATCTAACTGACACCTTAGACAATATCGACACTACA
AACCAAACTTCCACGATTTTCCGTAACCATAAATTGATGAATAATGTTCTAATACACAAAACAGAATAAACTGATAACAACAAATCTATCACATAATCTAACTGACACCTTAGACAATATC
AACCAAACTTCCACGATTTTCCGTAACCATAAATTGATGAATAATGTTCTAATACACAAAACAGAATAAACTGATAACAACAAATCTATCACATAATCTAACTGACACCTTAGACAATATC

911      920       930       940       950       960       970       980       990       1000      1010      1020      1030      1040
GTCACATACCTCACTAACATAATACAAGTTAAAGTAACACATCACAACTATAACATAAACACCCACTAACAACAAACAACTAACCACTGACACCTAACATATTCTGACCACAGCTCTATATTACACCAAC

1041     1050      1060      1070      1080      1090      1100      1110      1120      1130      1140      1150      1160      1170
CCCAATCGAACCCCACCTCCCCGTGACAACCCCCGATTTTGGCAAAATAATGTACGGGGGAGATGCATGAATTTCCGGTCCCAAATTTGAACGCCCCTCCCAAAACCGAACTTCCACGAATTTTCTAACC

1171     1180      1190      1200      1210      1220      1230      1240      1250      1260      1270      1280      1290      1300
CGCATCTTACCCATATCAACCTAAATCAAACCATAACTTTCATAATATTATAAACATAATAAACATATAACCACAATAAACACAGTCTCACTTCCAACCTAAACTTTTCTATCACCATCGTTCTGTATTA

1301     1310      1320      1330      1340      1350      1360      1370      1380      1390      1400      1410      1420      1430
ACTATCATAATAAACATTCACAAGATACAAAATAACTAAACTGTCTATACCTATATCTATCCAACTCACCCGCAACCAAGCCCTATATTACACCAACCCCAATCGAACCCCACCTCCCCGAAAATCCCGC

1431     1440
AAATCCAGTA
```

Tc_minicircle_Y_4.42

```
1        10        20        30        40        50        60        70        80        90        100       110       120       130
ATCTTGTTTTGGGAGGGGCGTTCAAATTTGGGGCCGGAAATTCATGCATCTCCCCCGTACATTATTTGGTCCGAAATCCTGAATTTTACGGGGAGGTGGGGTTCGATTGGGGTTGGTGTAATATAGGCAG

131      140       150       160       170       180       190       200       210       220       230       240       250       260
TGGGTGTCGGTGGTATAGCATTGTGATTGTTATGTTATGATGATTAGGTTTGTGATGTTGGTATAGTACATTGAAGTGATGATTGAGTATACATCGAGGTGAGGGTATAGAGTGAGTAGTAGTATGTTCT

261      270       280       290       300       310       320       330       340       350       360       370       380       390
AGTTTGAGATATACTTTATTATTTGATTTATATATTTGTTATAATTTGTCTGGTTAATGGTTGTCATTGGTTAAATGGTGGTTCAGAAAATTGTGGAAATTTGGTTTTGGGAGGGGCGTTCAAATTTGGG

391      400       410       420       430       440       450       460       470       480       490       500       510       520
GCCGGAAATTCATGCATCTCCCCCGTACATTATTTGTCTGAAAATGGGGGTTGTCACGGGGAGGTGGGGTTCGATTGGGGTTGGTGTAATATAGCATCTGGTTGTTGGAGGGGTGGTTTAGGAGTATGCG

521      530       540       550       560       570       580       590       600       610       620       630       640       650
TATAGAGGGTGTAGTGTATAGTTATTTAAAGATGTTGGTTATCGTAGTTGATAATGTTGATAATTTGTTGTGACTAGATGAGTGGTGGTTTTATGTTGTACTTAGTGAATTGTTATTTATGTTTTTGTTT
                                                                                                                     TATTTATGTTTTTGTTT
                                                                                                                     TATTTATGTTTTTGTTT

651      660       670       680       690       700       710       720       730       740       750       760       770       780
GATGACTAGAATAGATTTATATTATAATGATAAGGTTGTAGTAGGTCTGGAAAAAGTGGGAAACTTGGTTTTGGGAGGGGCGTTCAACTTTTGGGGGCCAAATTCATGCATCTCCCCCGTACATTATTTT
GATGACTAGAATAGATTTATATTATAATGATAAGGTTGTAGTAGGTCTGGAAAAAGTGGGAAACTTGGTTTTGG
GATGACTAGAATAGATTTATATTATAATGATAAGGTTGTAGTAGGTCTGGAAAAAGTGGGAAACTTGGTTTTGG

781      790       800       810       820       830       840       850       860       870       880       890       900       910
ATCGGATTTGCGATTTTTACGGGGAGGTGGGGTTCGATTGGGGTTGGTGTAATATAAGGTTTTGGATGGTAGAATGTGGATGGTGAGTTATTTATGAGTTGAGTTGTGTATAGTTCAGTTTGTATAGAGT

911      920       930       940       950       960       970       980       990       1000      1010      1020      1030      1040
AGTTTGTGATTGTTTATGATAGAGCTAGTACACCAAAGTGATGATAGTTGTTAGAGGTAAAGAAGTGAAATTTGTTTGGATATGTATTGAGATGTTGTATAGATCTTAATTTGTATAATGTTGTTGAGGT

1041     1050      1060      1070      1080      1090      1100      1110      1120      1130      1140      1150      1160      1170
TGGAAGTTAAATTTGGGGGTTAGAAAATAGCGTAAAGTTGGTTTTGGGAGGGGCGTTCAACTTTTGGGGGCCAAATTCATGCATCTCCCCCGTACATTATTTTGGCGATTTTGCGATTTTATTGGTGAGG

1171     1180      1190      1200      1210      1220      1230      1240      1250      1260      1270      1280      1290      1300
TGGGGTTCGATTGGGGTTGGTGTAATATAACCAGAATGATTATAAGTTGGTTAGTATAGATTTATTATTTATTGTGTTTGTATGATGTGATTGAACCGCATGAGAGTTATAGATGATAGTGAAGATAGTT

1301     1310      1320      1330      1340      1350      1360      1370      1380      1390      1400      1410      1420      1430
AAAGATTGTTGGTGAAGTTTGATTATGAATTCTGTCGTATGTTATTATAATATGGTAGGGTCAATGTTGGTGTTGTGAGTTAGGGTTGTGGTGGTTGCATTATGGTAGAAATGGGTTATGAAAATTTGGC

1431
AAA
```

Tc_minicircle_Y_4.114

```
1       10        20        30        40        50        60        70        80        90        100       110       120       130
|--------+---------+---------+---------+---------+---------+---------+---------+---------+---------+---------+---------+---------|
ATCGGCAAAATAATGTACGGGGGAGATGCATGAATTCTGGCCCTGAAAGTTGAACGCCCCTCCCAAAACAAGAATTTGCTGAATTTCTGAACCATTATCTTAACCCAAAACCATAACCACACCAACCACA

..................................................................................................................................

131     140       150       160       170       180       190       200       210       220       230       240       250       260
|--------+---------+---------+---------+---------+---------+---------+---------+---------+---------+---------+---------+---------|
CCAACCATTAACACAAGTATACCAGTATAGCATTAACATATTATACACTAAACAAACAAATTATCAAACTTACTCAACTCACTAACAAACGTACCTTATTCATTCACTAGCGTAACATCACCTATAACAT

..................................................................................................................................

261     270       280       290       300       310       320       330       340       350       360       370       380       390
|--------+---------+---------+---------+---------+---------+---------+---------+---------+---------+---------+---------+---------|
ATACAAAACTATAATTAGTAAATAAACTCTTTATACTCCAATCCTTATATTACACCAACCCCAATCGAACCCCACCTCCCCGACAAAATCGCAAAATCGGCAAAATAATGTACGGGGGAGATGCATGAAT

..................................................................................................................................

391     400       410       420       430       440       450       460       470       480       490       500       510       520
|--------+---------+---------+---------+---------+---------+---------+---------+---------+---------+---------+---------+---------|
TCTGGCCCTGAAAGTTGAACGCCCCTCCCAAAACCAGAATTTCCGGAATTTTACAAACCCATTCACTTACATACCATACCCCACAACACAAACTAACCACCAAACACAAATATAACATAGAGCTATAACA

..................................................................................................................................

521     530       540       550       560       570       580       590       600       610       620       630       640       650
|--------+---------+---------+---------+---------+---------+---------+---------+---------+---------+---------+---------+---------|
TTATCAATACTAACTAACTACAACAAATTCAACTCTAAATCATTCTTAACCAACTCATTACATAACACACAACCATTCTCTAAATCCAACTCAACATACAAAATACATAGAATACATTTTAACCTTACCC
                                                                                                      ATACATAGAATACATTTTAACCTTACCC
......................................................................................................ATACATAGAATACATTTTAACCTTACCC

651     660       670       680       690       700       710       720       730       740       750       760       770       780
|--------+---------+---------+---------+---------+---------+---------+---------+---------+---------+---------+---------+---------|
ATGCCAATCCCTATATTACACCAACCCCAATCGAACCCCACCTCCGCGTGACAACCCCCAAATTTGCCAAAATAATGTACGGGGGAGATGCATGAATTTTCCGGTCCAAATTTGAACGCCCCTCCCAAAA
ATGCCAATCCCTATATTACACCAACCCCAATCGAACCCCACCTCCGCGTGACAACCCCCAAATTTGCCAAAATAATGTACGGGGGAGATGCATGAATTTTCCGGTCCAAATTTGAACGCCCCTCCCAAAA
ATGCCAATCCCTATATTACACCAACCCCAATCGAACCCCACCTCCGCGTGACAACCCCCAAATTTGCCAAAATAATGTACGGGGGAGATGCATGAATTTTCCGGTCCAAATTTGAACGCCCCTCCCAAAA

781     790       800       810       820       830       840       850       860       870       880       890       900       910
|--------+---------+---------+---------+---------+---------+---------+---------+---------+---------+---------+---------+---------|
CATAATTTTCTGAATTTTCTTACCTCCAAAATACACAATAATATTATACACAATACCCTTCCATTACCTACCAAACAACTAACCCTATCTACCAAGCATAAACATTATTATACAATACACAGCTAACTAA
CATAATTTTCTGAATTTTCTTACCTCCAAAATACACAATAATATTATACACAATACCCTTCCATTACCTACCAAACAACTAACCCTATCT
CATAATTTTCTGAATTTTCTTACCTCCAAAATACACAATAATATTATACACAATACCCTTCCATTACCTACCAAACAACTAACCCTATCT........................................

911     920       930       940       950       960       970       980       990       1000      1010      1020      1030      1040
|--------+---------+---------+---------+---------+---------+---------+---------+---------+---------+---------+---------+---------|
TACAAAGACACTAACAAACTATCTTCTCTCAACTTTCTATTCCTCTTCTGTTTGTCCTCATACACATCATAAACATATTCTATCAACTATACAACTGTAATCCACCACTCCTATATTACACCAACCCCAA

..................................................................................................................................

1041 1050       1060      1070      1080      1090      1100      1110      1120      1130      1140      1150      1160      1170
|--------+---------+---------+---------+---------+---------+---------+---------+---------+---------+---------+---------+---------|
TCGAACCCCACCTCCCCGTGACAACCCCCAATTTTACCGAAATAATGTACGGGGGAGATGCATGAATTTTCCGGTCCAAATTTGAACGCCCCTCCCAAAATCAGATTTTCCTAATTTTTCGTAATCAATT

..................................................................................................................................

1171 1180       1190      1200      1210      1220      1230      1240      1250      1260      1270      1280      1290      1300
|--------+---------+---------+---------+---------+---------+---------+---------+---------+---------+---------+---------+---------|
TTATCACACGTAACCAATATTCAACCTCAACACAGACTACCACAACCCCACCAAACAATAATTTTATAAATATCAAATATATTCTATAACAACTTCAACTATACAAGAATCTACAACATACTACAACCCA

..................................................................................................................................

1301 1310       1320      1330      1340      1350      1360      1370      1380      1390      1400      1410      1420      1430
|--------+---------+---------+---------+---------+---------+---------+---------+---------+---------+---------+---------+---------|
TAATGTTTGTGCAGTCAACTACTCAAGTCACACAACCTCATTAACTATACATACGACATTAATGTACCTTCAGCCTCCATGCTATATTACACCAACCCCAATCGAACCCCACCTCCCTGACAAAATCACA

..................................................................................................................................

14[illegible]31
||
AA

..
```

Tc_minicircle_Y_4.115

```
1        10        20        30        40        50        60        70        80        90        100       110       120       130
AATGTACGGGGGAGATGCATGAATTTTCCGGTCCAAATTTGAACGCCCCTCCCAAAACCAGGAATCTGCGTAATTTCGTAACCTATAATCTACCAATATCAACACCATAACACACCCCTCATTCAAACAT

131      140       150       160       170       180       190       200       210       220       230       240       250       260
TATACTTTAACAAAACAATAACTACAAACTAATACACGTCAAATTATCTTTACACACTCATTCATATCCTATCTTGTGCAACTCATATTACATCATCATGATCACAGTAACAAAACTATACTATAGCTCT

261      270       280       290       300       310       320       330       340       350       360       370       380       390
CAACTAAAATATTATCCATACCCACTGATGCTATATTACACCAACCCCAATCGAACCCCACCTCCCCGACAAAATGCAAAAATCAGCAAAATAATGTACGGGGGAGATGCATGAATTCTGGCCCTGAAAG

391      400       410       420       430       440       450       460       470       480       490       500       510       520
TTGAACGCCCCTCCCAAAACCAGGTTTCCACCAAATTTCGTTCACCAAATTATACCAAACAAACACAACCAACCACACCCCATTATCACCCTACTACACCTCCAAGTATAAAATTATTATAACATATATC

521      530       540       550       560       570       580       590       600       610       620       630       640       650
AATTCATAATCAATCAACATACCAAATAACTCTGCACTACTTACTATACCCTCACAATGAATTCACTGTCTACAATATATCAAACAATTCACCTAACCTACTAAATCTTATAACACCATCCACAACAATC
                                                                                                                        CACAACAATC
........................................................................................................................CACAACAATC

651      660       670       680       690       700       710       720       730       740       750       760       770       780
CCTATATTACATCAACCCCAATCGAACCCCACCTCCCCGTGACAACCCCCATTTTCAGCCAAATAATGTACGGGGGAGATGCATGAATTTTCCGGTCCAAATTTGAACGCCCCTCCCAAAACCAAGATTT
CCTATATTACATCAACCCCAATCGAACCCCACCTCCCCGTGACAACCCCCATTTTCAGCCAAATAATGTACGGGGGAGATGCATGAATTTTCCGGTCCAAATTTGAACGCCCCTCCCAAAACCAAGATTT
CCTATATTACATCAACCCCAATCGAACCCCACCTCCCCGTGACAACCCCCATTTTCAGCCAAATAATGTACGGGGGAGATGCATGAATTTTCCGGTCCAAATTTGAACGCCCCTCCCAAAACCAAGATTT

781      790       800       810       820       830       840       850       860       870       880       890       900       910
CCGGAAAAATTCTGAACCTATCTCTGAACCCATAACACCTAACCGTAAATTCTTATAATACAACATACTCATTAACTACATACATTAACAAACAAACATACTCACACTATCTCTACTCTCTTCAGTGTATC
CCGGAAAAATTCTGAACCTATCTCTGAACCCATAACACC
CCGGAAAAATTCTGAACCTATCTCTGAACCCATAACACC...........................................................................................

911      920       930       940       950       960       970       980       990       1000      1010      1020      1030      1040
CTCTACCAATCAAATAATAACAAACCATAACATACATAACATAAACTACATACATAATACATATACCATACCACACCTACCAACCCACAGGCTATATTACACCAACCCCAATCGAACCCCCACCTCCCCG

1041 1050      1060      1070      1080      1090      1100      1110      1120      1130      1140      1150      1160      1170
ACAAAATGCGAAAATCAGCAAAATAATGTACGGGGGAGATGCATGAATTCTGGCCCCCAAAGTTGAACGCCCCTCCCAAAACCAAATCCCCCGCAAATTTTATACCCATAAATTCACAACCTAACATACA

1171 1180      1190      1200      1210      1220      1230      1240      1250      1260      1270      1280      1290      1300
ACCTACCTCAACCACATTCCAAACCATACACCTTACCAATACCACAACTATACAAATACACTCAATACTATCAAATAATAAACACTACACAAAGTAACAACAACAACTCATAAAACTAAATCATACCATC

1301 1310      1320      1330      1340      1350      1360      1370      1380      1390      1400      1410      1420
TCGACACCAACTATCATATAAACCATACAAACTACAACGCTATAATAAACTCAAACCCTATATTACACCAACCCCAATCGAACCCCACCTCCCCGTGACAACCCCCATTTTCCAGCAAAT
```

Tc_minicircle_Y_3.4

```
1        10        20        30        40        50        60        70        80        90        100       110       120       130
|--------+---------+---------+---------+---------+---------+---------+---------+---------+---------+---------+---------+---------|
TTGTTGTGTACAGTATATGTATGTTAATGTATGAGGGTACTGGTATAGGTTTTGGGTTTGTGTATGGGTGTATAATTGAATTGTTGGTAGAATTGGTTACGAAAATTTGTGGAAACTGTGGTTTTGGGAG

..................................................................................................................................

131      140       150       160       170       180       190       200       210       220       230       240       250       260
|--------+---------+---------+---------+---------+---------+---------+---------+---------+---------+---------+---------+---------|
GGGCGTTCAAATTTGGGGCCGAAAAATCATGCATCTCCCCCGTACATTATTTCCGGGATTTTATGGAAATTTCGGGGAGGTGGGGGTTCGATTGGGGTTGGTGTAATATAAGGCTGGGGTAGATTATGTA

..................................................................................................................................

261      270       280       290       300       310       320       330       340       350       360       370       380       390
|--------+---------+---------+---------+---------+---------+---------+---------+---------+---------+---------+---------+---------|
TTAAGACTGAGTAGATGTTGTAAGATTATGTAGTGTGATTTTGTGATGTATGCAGAGATAAAACAAGAATGGTGAGAGTGATAGTAGTATAGTTCTTTGTGTTCTTTGATAGTAGATAGTATATGTTATA

..................................................................................................................................

391      400       410       420       430       440       450       460       470       480       490       500       510       520
|--------+---------+---------+---------+---------+---------+---------+---------+---------+---------+---------+---------+---------|
TTATAAATGTTAATAAGGTGGGTGCTGTTAGTTTTGGTAATGGTATAGGATCAACATAAAATAGTGTCCTGAAATTTCGCGGATTCTTGGTTTTGGGAGGGGCGTTCAAATTTGGACCGGAAAATTCATG
                                                                            TCGCGGANTCTTGGTTTTGGGAGGGGCGTTCAAATTTGGACCGGAAAATTCATG
............................................................................TCGCGGAnTCTTGGTTTTGGGAGGGGCGTTCAAATTTGGACCGGAAAATTCATG

521      530       540       550       560       570       580       590       600       610       620       630       640       650
|--------+---------+---------+---------+---------+---------+---------+---------+---------+---------+---------+---------+---------|
CATCTCCCCCGTACATTATTTTGCAATTTTAGGGGGTTGTCACGGGGAGGTGGGGTTCGATTGGGGTTGGTGTAATATAGGATACGAGCTATGAGATGTTTGTTTAGTTGTTAGTTAGTGATTGTATGGT
CATCTCCCCCGTACATTATTTTGCAATTTTAGGGGGTTGTCACGGGGAGGTGGGGTTCGAT
CATCTCCCCCGTACATTATTTTGCAATTTTAGGGGGTTGTCACGGGGAGGTGGGGTTCGAT.....................................................................

651      660       670       680       690       700       710       720       730       740       750       760       770       780
|--------+---------+---------+---------+---------+---------+---------+---------+---------+---------+---------+---------+---------|
ATTGAGTTTAGGTGAGATTGCGTACATAATAGTTTGTATTGGATAGATTTGAGGTGTTAGTTTAAGTATTGAAGTTATAGATTTGATTTGTATGTTTATGTGTTAGGAGGGTGTATGATTGTGCGGTGTG

..................................................................................................................................

781      790       800       810       820       830       840       850       860       870       880       890       900       910
|--------+---------+---------+---------+---------+---------+---------+---------+---------+---------+---------+---------+---------|
AGTTGGTTGTGTGGTATGTGGTGAATTGGAGGTTAAAAATTTCCTGGAAACCTGGTTTTGGGAGGGGCGTTCAAATTTGGACCGGAAAATTCATGCATCTCCCCCGTACATTATTTGGTCGAAAATGGGG

..................................................................................................................................

911      920       930       940       950       960       970       980       990      1000      1010      1020      1030      1040
|--------+---------+---------+---------+---------+---------+---------+---------+---------+---------+---------+---------+---------|
GTTGTCACGGGGAGGTGGGGTTCGATTGGGGTTGGTGTAATATAGGATACGAGTTATGATTATGATTATTTAGTTTATGTGGATATTGATGTTTACGTGTGAGGCAATGTACATGAGTTTGTGTTGTATA

..................................................................................................................................

1041  1050      1060      10701074
|--------+---------+---------+---|
CTCGGTGTGAATTTGATTTTGATTTGATTTGAAT

..................................
```

Tc_minicircle_Y_4.69

```
1       10        20        30        40        50        60        70        80        90        100       110       120       130
|--------+---------+---------+---------+---------+---------+---------+---------+---------+---------+---------+---------+---------|
GTTCCCAGAATTCTAGAGATCCAAATTTAACATACATACTCATCCACCAAACCACAGAATTACCAACATAACCTATACCACCACTAACAACTTAATAATATAATATCTTAATAATACACTAACATCAAAT

..................................................................................................................................

131     140       150       160       170       180       190       200       210       220       230       240       250       260
|--------+---------+---------+---------+---------+---------+---------+---------+---------+---------+---------+---------+---------|
CAATACAACTCTCATCATCTTTCTCACACCTTAGAAACTCACACTGCCTCCTAACACATATCAAACTAATCATCCACTAATAATAAAATCAGAAAATCATAACATAATAAATGCTATATTACACCAACCC

..................................................................................................................................

261     270       280       290       300       310       320       330       340       350       360       370       380       390
|--------+---------+---------+---------+---------+---------+---------+---------+---------+---------+---------+---------+---------|
CAATCGAACCCCACCTCCCCGTGACAACCCCTGATTTTGGGGAAATAATGTACGGGGGAGATGCATGAATTTTCCGCCCCAAATTTGAACGCCCCTCCCAAAACCGAATTTTCTGAAATTTCCGTAACCT

..................................................................................................................................

391     400       410       420       430       440       450       460       470       480       490       500       510       520
|--------+---------+---------+---------+---------+---------+---------+---------+---------+---------+---------+---------+---------|
ATTTTATCAACACAACATCCTGTGCACACTAACCCTACCACACACCACACTATACTTATCAAATTATATACATTAACAATATCAAACTTAAATATACAACATATTACAAATCACTACGCACAATTCAAAT

..................................................................................................................................

521     530       540       550       560       570       580       590       600       610       620       630       640       650
|--------+---------+---------+---------+---------+---------+---------+---------+---------+---------+---------+---------+---------|
CACATTCTATTATGTATCCTAACCACTATCAAACTAAATTCAAACAAATAACTAAAATTATAAACTACCAATAACACTATATTACACCAACCCCAATCGAACCCCACCTCCCCGTGACAACCCCCAAATT
                                       TCAAACAAATAACTAAAATTATAAACTACCAATAACACTATATTACACCAACCCCAATCGAACCCCACCTCCCCGTGACAACCCCCAAATT
.......................................TCAAACAAATAACTAAAATTATAAACTACCAATAACACTATATTACACCAACCCCAATCGAACCCCACCTCCCCGTGACAACCCCCAAATT

651     660       670       680       690       700       710       720       730       740       750       760       770       780
|--------+---------+---------+---------+---------+---------+---------+---------+---------+---------+---------+---------+---------|
TGGTCAAATAATGTACGGGGGAGATGCATGAATTTTCCGCCCAAAATCTGAACGCCCCTCCCAAATCCAGGTTTTCTGAAATTTCCGTAACCTATTTTATCCTGGAATACACAACAACATTCAACCAAAC
TGGTCAAATAATGTACGGGGGAGATGCATGAATTTTCCGCCCAAAATCTGAACGCCCCTCCCAAATCCAGGTTTTCTGAAATTTCCGTAACCTATTTTATCCTGGAATACACAACAACATTCAACCAAAC
TGGTCAAATAATGTACGGGGGAGATGCATGAATTTTCCGCCCAAAATCTGAACGCCCCTCCCAAATCCAGGTTTTCTGAAATTTCCGTAACCTATTTTATCCTGGAATACACAACAACATTCAACCAAAC

781     790       800       810       820       830       840       850       860       870       880       890       900       910
|--------+---------+---------+---------+---------+---------+---------+---------+---------+---------+---------+---------+---------|
CCCTACCACACCACAAACCATAGTAACCATATAAAATAAATAAATCAATAAACATTAGTAACAAACTTTAACTATGTATACCGAACACTATGCAAACTCTCTACATACCTATACGTTAGACATCACACAT
CCCTACCACACCACAAACCATAGTAACCATATAAAATAAATAAATCAATAAACATTAGTAACAAACTTTAACTATGTATACCGAACACTATGCAAAC
CCCTACCACACCACAAACCATAGTAACCATATAAAATAAATAAATCAATAAACATTAGTAACAAACTTTAACTATGTATACCGAACACTATGCAAAC.................................

911     920       930       940       950       960       970       980       990       1000      1010      1020      1030      1040
|--------+---------+---------+---------+---------+---------+---------+---------+---------+---------+---------+---------+---------|
CACATACTCCAAATCAGAAATACCATCTAACTAAACCAATAACTACACAGTCTATATTACACCAACCCCAATCGAACCCCACCTCCCCGACAAAATCACAAAATAGCCAAAATAATGTACGGGTGAGATG

..................................................................................................................................

1041  1050      1060      1070      1080      1090      1100      1110      1120      1130      1140      1150      1160      1170
|--------+---------+---------+---------+---------+---------+---------+---------+---------+---------+---------+---------+---------|
CATGAATTCGGGCCCTGAAAGTTGAACGCCCCTCCCAAAACAAGAGTTCCGCCATTTTCCGTAGTCAAATTTTAACCATGTTATTGTTATAAACATACACTTAACAACATTAGCAATCTAAATCACCTTA

..................................................................................................................................

1171  1180      1190      1200      1210      1220      1230      1240      1250      1260      1270      1280      1290      1300
|--------+---------+---------+---------+---------+---------+---------+---------+---------+---------+---------+---------+---------|
CACACCAAACTCATATTCTACTAGCTGTCTATCTCTCACTTCATACTATCTAACAAACTCAATCAAAGTGTATAAACAACCATAAACTACAAACATACTATCAAAATAACATAACCCTTAACACTCTACC

..................................................................................................................................

1301  1310      1320      1330      1340      1350      1360      1370      1380      1390      1400      1410      1420      1430
|--------+---------+---------+---------+---------+---------+---------+---------+---------+---------+---------+---------+---------|
AACACAGGTACTATATTACACCAACCCCAATCGAACCCCACCTCCCCGAAAATTCCACCAATTTCCCAAAATAATGTACGGGGGAGATGCATGAAAATCCGGCCCCAAATTTGAACGCCCCTCCCAAAAC

..................................................................................................................................

143[illegible]
|--|
AGGA

....
```
