# Supplementary material for: The Complete Mitochondrial DNA of Trypanosoma cruzi: Maxicircles and Minicircles
Source: Front Cell Infect Microbiol. 2021 Jun 29;11:672448. doi: 10.3389/fcimb.2021.672448 (PMC8277381; doi:10.3389/fcimb.2021.672448)
Supplement: Supplementary file 6 [file Table_1.docx]

**Supplementary Table S1**. Primer sequences of Y strain minicircles. Forward (F) and reverse (R) sets of primers are indicated.

| Minicircle | Primer sequences | |
| --- | --- | --- |
| Tc_minicircle_Y_4.155 | F | 5’-GTGAGTGTGTTGGTTACAGGATAG-3’ |
|  | R | 5’-CACCCATAAATGCTATATTACACC-3’ |
| Tc_minicircle_Y_4.98 | F | 5’-GTTACGGTGTTGGTGTATGC-3’ |
|  | R | 5’-CAGTAACCATAACCACCACATC-3’ |
| Tc_minicircle_Y_4.128 | F | 5’-TCATACACGTTCTCTACCACA-3’ |
|  | R | 5’-GTAGGGTGTGCTTGTGTTG-3’ |
| Tc_minicircle_Y_4.12 | F | 5’-TCAACCTATCCAACTTTATATTCTG-3’ |
|  | R | 5’-GTCGATATTGTCTAAGGTGTCAG-3’ |
| Tc_minicircle_Y_4.42 | F | 5’-GTTGATAATTTGTTGTGACTAGATG-3’ |
|  | R | 5’-CACCATCCACATTCTACCAT-3’ |
| Tc_minicircle_Y_4.114 | F | 5’-CATTACATAACACACAACCATTC-3’ |
|  | R | 5’--TTATGCTTGGTAGATAGGGTTAG3’ |
| Tc_minicircle_Y_4.115 | F | 5’-CCTCACAATGAATTCACTGTC-3’ |
|  | R | 5’-ATTTACGGTTAGGTGTTATGGG-3’ |
| Tc_minicircle_Y_3.4 | F | 5’-GTGCTGTTAGTTTTGGTAATGG-3’ |
|  | R | 5’-CGCAATCTCACCTAAACTCA-3’ |
| Tc_minicircle_Y_4.69 | F | 5’-ACGCACAATTCAAATCACA-3’ |
|  | R | 5’-GAGTTTGCATAGTGTTCGGTAT-3’ |
