# Supplementary material for: The Complete Mitochondrial DNA of Trypanosoma cruzi: Maxicircles and Minicircles
Source: Front Cell Infect Microbiol. 2021 Jun 29;11:672448. doi: 10.3389/fcimb.2021.672448 (PMC8277381; doi:10.3389/fcimb.2021.672448)
Supplement: Supplementary file 7 [file Table_2.docx]

**Supplementary Table S2**. Genes and their location in the *T. cruzi* Bug2148 and Y strains maxicircles.

| **Coordinates (5'-3')** | | |
| --- | --- | --- |
| **Gene*** | **Bug2148 strain** | **Y strain** |
| ND5 | 32501-34100 | 15326-16925 |
| RPS12 | 34122-34309 | 15117-15304 |
| ND3 | 34386-34578 | 14848-15040 |
| ND4 | 34572-35883 | 13543-14854 |
| MURF2_gRNA | 35881-35921 | 13505-13545 |
| CR4 | 35985-36193 | 13233-13441 |
| COI | 36245-37894 | 11532-13181 |
| MURF2 | 37885-38937 | 10489-11541 |
| COII+gRNA | 38948-39592 | 9834-10478 |
| NDI | 39606-40547 | 8879-9820 |
| CR3 | 40541-40662 | 8764-8885 |
| MURF1 | 40649-41989 | 7437-8777 |
| ATPase_6 | 42046-42375 | 7051-7380 |
| Cyb | 42417-43495 | 5931-7009 |
| COIII | 43568-43987 | 5439-5858 |
| ND7 | 44061-44807 | 4619-5365 |
| MURF5 | 44883-45143 | 4283-4543 |
| ND9 | 45137-45486 | 3940-4289 |
| ND8 | 45549-45828 | 3598-3877 |
| 9S_rRNA | 45872-46481 | 2945-3554 |
| 12S_rRNA | 46525-47684 | 1742-2901 |
|  |  |  |
| *The names for individual genes are those used for Ruvalcaba-Trejo and Sturm (2011). | | |
